# A multi-component health literacy intervention addressing needs of kidney patients and professionals

**DOI:** 10.1093/eurpub/ckac129.380

**Published:** 2022-10-25

**Authors:** M Boonstra, SA Reijneveld, R Westerhuis, JM Tullius, JPM Vervoort, G Navis, AF de Winter

**Affiliations:** 1 Department of Health Sciences, University Medical Centre Groningen, Groningen, Netherlands; 2 Department of Nephrology, University Medical Centre Groningen, Groningen, Netherlands

## Abstract

**Background:**

Limited health literacy (LHL) is associated with faster kidney deterioration. To maintain kidney function, health care professionals (HCPs) promote self-management. However, in chronic kidney disease (CKD) care organizations, patients with LHL report multiple communication and self-management barriers. The need for health literacy responsive organizations, and multi-component interventions targeted at patients and HCPs, is recognized. We aimed to determine the objectives and strategies of such an intervention, and to co-create and evaluate it.

**Methods:**

First, we performed a longitudinal qualitative study among CKD patients with LHL (n = 24) and HCPs (n = 37) from general practices and nephrology clinics to assess needs and barriers, and to identify promising intervention objectives. Second, we included patients (n = 19), HCPs (n = 15), educators (n = 3) and students (n = 4) in an Intervention Mapping (IM) process to co-create and evaluate the intervention.

**Results:**

Grip on Your Kidneys (GoYK) addresses the competences of patients with LHL to self-manage CKD on the long-term, and the communication competences of patients and HCPs. Based upon patients’ preferences, GoYK encompasses a visually attractive website and brochures to optimize self-management and a card to contribute to consultations. With HCPs, we developed a training to optimize their competences to support patients with LHL. Evaluation revealed the intervention was useful, comprehensive and fitting needs.

**Conclusions:**

Mapping the needs of CKD patients and HCPs, combined with a process of intervention co-creation, resulted in a multi-component, positively evaluated intervention. Implementation of this type of interventions in organizations is promising to improve care for patients with LHL. However, the effectiveness of these interventions requires further assessment.

